# Cluster Analysis of Primary Care Physician Phenotypes for Electronic Health Record Use: Retrospective Cohort Study

**DOI:** 10.2196/34954

**Published:** 2022-04-15

**Authors:** Allan Fong, Mark Iscoe, Christine A Sinsky, Adrian D Haimovich, Brian Williams, Ryan T O'Connell, Richard Goldstein, Edward Melnick

**Affiliations:** 1 National Center for Human Factors in Healthcare MedStar Health Washington, DC United States; 2 Department of Emergency Medicine Yale School of Medicine New Haven, CT United States; 3 American Medical Association Chicago, IL United States; 4 Northeast Medical Group Yale New Haven Health Stratford, CT United States

**Keywords:** electronic health record, phenotypes, cluster analysis, unsupervised machine learning, machine learning, EHR, primary care

## Abstract

**Background:**

Electronic health records (EHRs) have become ubiquitous in US office-based physician practices. However, the different ways in which users engage with EHRs remain poorly characterized.

**Objective:**

The aim of this study is to explore EHR use phenotypes among ambulatory care physicians.

**Methods:**

In this retrospective cohort analysis, we applied affinity propagation, an unsupervised clustering machine learning technique, to identify EHR user types among primary care physicians.

**Results:**

We identified 4 distinct phenotype clusters generalized across internal medicine, family medicine, and pediatrics specialties. Total EHR use varied for physicians in 2 clusters with above-average ratios of work outside of scheduled hours. This finding suggested that one cluster of physicians may have worked outside of scheduled hours out of necessity, whereas the other preferred ad hoc work hours. The two remaining clusters represented physicians with below-average EHR time and physicians who spend the largest proportion of their EHR time on documentation.

**Conclusions:**

These findings demonstrate the utility of cluster analysis for exploring EHR use phenotypes and may offer opportunities for interventions to improve interface design to better support users’ needs.

## Introduction

As of 2021, the vast majority of US office-based physicians used an electronic health record (EHR) [1]. The transition from paper to electronic records has many potential benefits but has also introduced new burdens. Furthermore, EHR use dominates clinical time [2] and is associated with burnout [3-5]. Despite the ubiquity of EHRs, patterns of clinician use are poorly characterized.

A 2019 survey study of clinicians reported widely divergent, subjective experiences with their EHR use and found that individual user differences accounted for over half of the variation in EHR use [6]. User-level variation can be due to disparities in proficiency that could potentially be remedied with appropriate training [7-10]. Emerging evidence suggests there are elements aside from proficiency that differentiate EHR users. For example, recent cross-sectional analyses of ambulatory care physicians’ EHR use have found significant differences in time spent on EHRs based on gender [11,12], specialty [12,13], and country [14].

Audit logs offer a wealth of information derived from granular observations of users’ EHR actions [15,16]. For example, research using log data has demonstrated associations between physicians’ EHR activities and vendor-defined metrics of efficiency [17] and that efficiency varied based on physicians’ years of experience and shift type [18]. In this study, we propose to use audit log data for the de novo identification of EHR user types (ie, EHR use phenotypes). Phenotype was first introduced by Richesson et al [19] as a biological concept to describe a set of observable biological traits. In the context of EHR use measures, phenotype will be used to describe observable use patterns across gender and specialty differences as defined by an unsupervised clustering approach called affinity propagation. First, 5 EHR use measures will be standardized using z-scores, which will then be used to calculate the similarities between physicians. A grid search and algorithm constraints will then be used to identify optimal clusters across a cohort of ambulatory care physicians.

## Methods

### Study Setting and Data Sources

This study retrospectively examined EHR log data of nontrainee, primary care physicians employed by a large ambulatory practice network (Northeast Medical Group) in northeastern United States (Connecticut, New York, and Rhode Island) between March 2018 and February 2020. Physicians were included if they specialized in general internal medicine, family medicine, or general pediatrics.

### Ethics Approval

All data were anonymized, with the investigators blinded to the participants’ identities. The study protocol was approved by Northeast Medical Group’s Institutional Review Board (IRB number 2000026556).

### EHR Use Measures

We retrieved data from the Epic Signal platform (Epic Systems) stratified by month and derived 5 proposed, time-based core EHR use measures normalized to 8 hours of scheduled patient time (Table 1) [20]. The first measure is EHR-Time_8_, defined as the time a physician spends on EHRs (both during and outside of scheduled patient hours) [20]. The second measure is work outside of work (WOW_8_), not to be confused with WOW carts (ie, workstations on wheels, a common industry term). WOW_8_ is defined as the time a physician works on EHRs outside of scheduled patient hours [20]. The third measure is Note-Time_8_, defined as the time a physician spends on documentation [20]. The fourth and fifth measures are IB-Time_8_ and Order-Time_8_, defined as the times a physician spends on inbox activities and on orders, respectively [20]. To account for relationships between EHR-Time_8_ and its composite measures, we reported the ratios of WOW_8_, Note-Time_8_, IB-Time_8_, and Order-Time_8_ to EHR-Time_8_, denoted as WOW-EHR, Note-EHR, IB-EHR, and Order-EHR, respectively. These measures (Table 1) were calculated and extracted from the Epic Signal platform, which have been validated and used in previous studies [20,21]. Each physician’s EHR use measures were averaged across study months to account for variation in metric calculations introduced by changes in measure definitions over time due to the vendor’s continuous quality improvement processes. For this analysis, we only considered physicians with valid metric months. Months with fewer than 30 clinical hours scheduled and less than 1 hour of EHR use were excluded from the analysis as invalid metric months. These thresholds were determined based on previous manual chart review validation and analysis of EHR vendor data [13].

**Table 1:** Electronic health record (EHR) use measures and definitions.

Measure	Definition
EHR-Time_8_	Time a physician spends on EHRs (both during and outside of scheduled patient hours) normalized to 8 hours of scheduled patient time
WOW-EHR	Ratio of EHR time that occurs during work outside of work (WOW_8_^a^) hours: WOW_8_/EHR-Time_8_
Note-EHR	Ratio of EHR time a physician spends on documentation: Note-Time_8_^b^/EHR-Time_8_
IB-EHR	Ratio of EHR time a physician spends on inbox (IB) activities: IB-Time_8_^c^/EHR-Time_8_
Order-EHR	Ratio of EHR time a physician spends on orders: Order-Time_8_^d^/EHR-Time_8_

^a^WOW_8_: work outside of work hours normalized to 8 hours of scheduled patient time.

^b^Note-Time_8_: note time hours normalized to 8 hours of scheduled patient time.

^c^IB-Time_8_: inbox time hours normalized to 8 hours of scheduled patient time.

^d^Order-Time_8_: order time hours normalized to 8 hours of scheduled patient time.

### Cluster Analysis

Clusters were required to include individuals from at least two primary care specialties. Moreover, we did not require that all individuals be assigned to a phenotype cluster while also seeking to minimize the total number of phenotypes. Affinity propagation, an algorithm that takes a set of pairwise similarities between data points and finds clusters on the basis of maximizing the total similarity between data points in a cluster, was used for phenotype discovery [22]. Affinity propagation has advantages over other clustering algorithms, such as not predefining a number of clusters. A major disadvantage of affinity propagation is its high computational cost and resource requirement; however, this approach was deemed feasible given this study’s sample size [22]. First, a standard z-score for each measure was calculated in order to center and scale the data. Similarities between data points were then calculated using Euclidean distance, which is defined for two 2D points as the length of the line formed by the two points. A grid search was then performed by varying the damping factor and preference from 0.5 to 1 and from 2 to 4, respectively, to identify the optimal clustering given the initial cluster conditions. Physicians in clusters that did not have representation from at least two specialties were excluded. Finally, physician gender and specialty distributions were described between clusters. All analyses were performed using Python software (version 3.7; Python Software Foundation) and scikit-learn (version 0.24; scikit-learn developers) [23].

## Results

### Identifying Clusters

**Figure 1:**
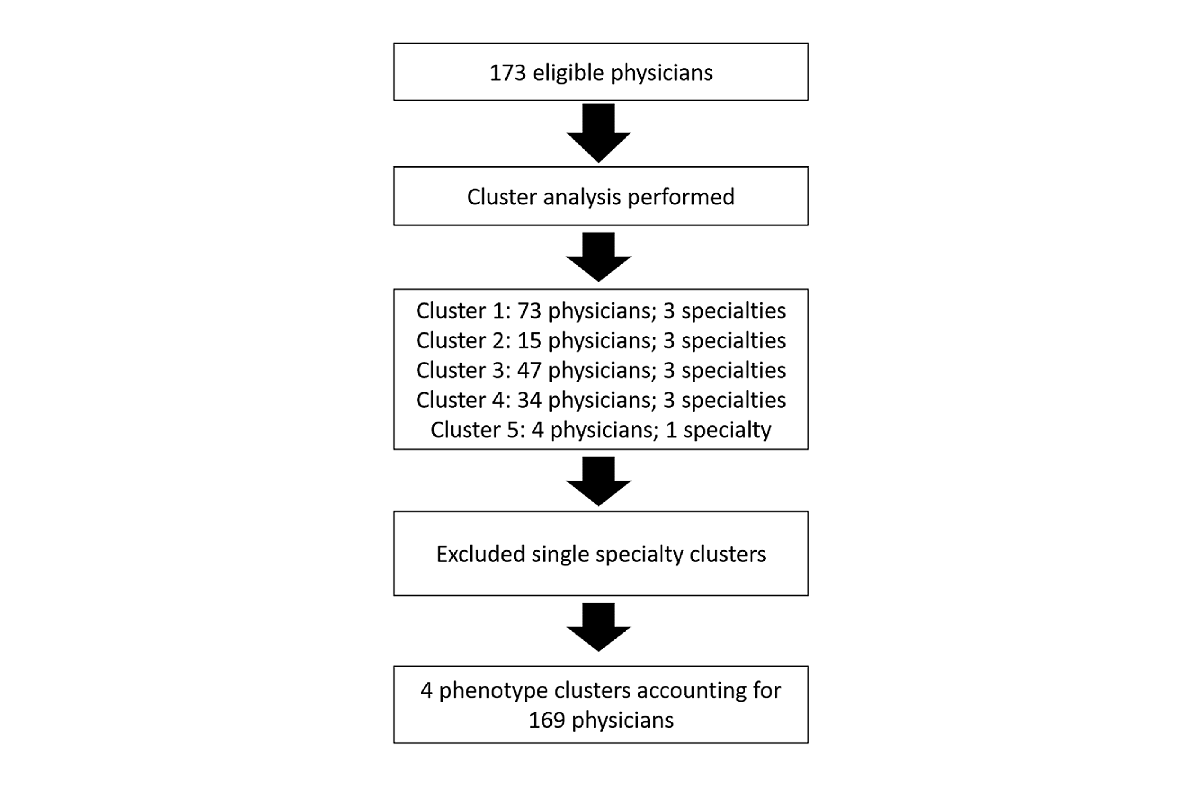
Summary of workflow and exclusion criteria.

Of 332 ambulatory, nontrainee physicians, 290 (87.3%) have valid month metrics. Of those, a further 173 (52.1%) eligible physicians were of the specialties of interest: 117 (67.6%) in internal medicine, 36 (20.8%) in family medicine, and 20 (11.6%) in pediatrics. Gender distribution of the eligible physicians was 47.4% (82/173) female and 52.6% (91/173) male. We identified 4 clusters that met our a priori defined clustering conditions, accounting for 97.7% (169/173) of eligible physicians (Figure 1).

### EHR Use Measures and Phenotypes Clusters

**Figure 2:**
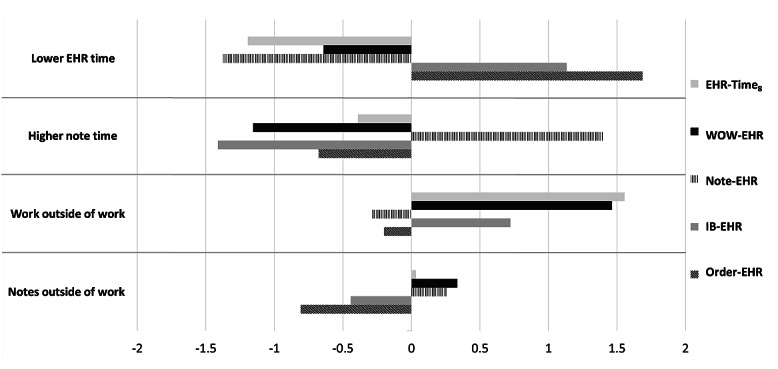
Z-scores for electronic health record (EHR) use measure across clusters. EHR-Time_8_: time a physician spends on EHRs normalized to 8 hours of scheduled patient time; IB-EHR: ratio of EHR time that a physician spends on inbox activities; Note-EHR: ratio of EHR time that a physician spends on documentation; Order-EHR: ratio of EHR time that a physician spends on orders; WOW-EHR: ratio of EHR time that occurs during work outside of scheduled hours.

The phenotype clusters are “Lower EHR time,” “Higher note time,” “Work outside of work,” and “Notes outside of work.” The EHR use measures across clusters are summarized in Table 2. There was a significant association between phenotype clusters and each EHR use measure: EHR-Time_8_ (Kruskal-Wallis *H*=72.7, *P*<.001), WOW-EHR (*H*=84.3, *P*<.001), Note-EHR (*H*=89.0, *P*<.001), IB-EHR (*H*=45.8, *P*<.001), and Order-EHR (*H*=46.8, *P*<.001). The z-scores for the measures are displayed in Figure 2 to illustrate the relative differences between clusters.

**Table 2:** Electronic health record (EHR) use measures by phenotype cluster.

Measure	Phenotype clusters, median (IQR)				
	Lower EHR time	Higher note time	Work outside of work	Notes outside of work	All
EHR-Time_8_^a^	4.62 (4.20-5.43)	5.81 (4.41-6.22)	6.83 (5.95-8.36)	5.90 (5.37-6.36)	5.62 (4.57-6.40)
WOW-EHR^b^	0.07 (0.04-0.12)	0.05 (0.03-0.07)	0.21 (0.17-0.26)	0.13 (0.10-0.19)	0.11 (0.06-0.19)
Note-EHR^c^	0.24 (0.20-0.28)	0.46 (0.43-0.49)	0.31 (0.27-0.36)	0.37 (0.33-0.40)	0.29 (0.24-0.38)
IB-EHR^d^	0.14 (0.12-0.18)	0.06 (0.05-0.08)	0.15 (0.11-0.17)	0.10 (0.08-0.12)	0.13 (0.09-0.16)
Order-EHR^e^	0.19 (0.17-0.24)	0.14 (0.12-0.17)	0.16 (0.14-0.18)	0.14 (0.12-0.17)	0.17 (0.14-0.20)

^a^EHR-Time_8_: time a physician spends on EHRs normalized to 8 hours of scheduled patient time.

^b^WOW-EHR: ratio of EHR time that occurs during work outside of scheduled hours.

^c^Note-EHR: ratio of EHR time that a physician spends on documentation.

^d^IB-EHR: ratio of EHR time that a physician spends on inbox activities.

^e^Order-EHR: ratio of EHR time that a physician spends on orders.

#### “Lower EHR Time” Cluster

The “Lower EHR time” cluster was the largest cluster, constituting 42.2% (73/173) of eligible physicians. Physicians in this cluster spent the least amount of time on EHRs (EHR-Time_8_: median 4.62, IQR 4.20-5.43). “Lower EHR time” cluster physicians had the lowest median Note-EHR ratio of 0.24 (IQR 0.20-0.28) and the second lowest median WOW-EHR ratio of 0.07 (IQR 0.04-0.12). They also had the highest median IB-EHR and Order-EHR ratios of 0.14 (IQR 0.12-0.18) and 0.19 (IQR 0.17-0.24), respectively.

#### “Higher Note Time” Cluster

“Higher note time” cluster physicians, constituting only 8.7% (15/173) of the total, had near-average normalized EHR time (EHR-Time_8_: median 5.81, IQR 4.41-6.22). Physicians in this cluster spent the largest proportion of their EHR time documenting notes (Note-Time: median 0.46, IQR 0.43-0.49) compared to physicians in other clusters. They also spent the lowest proportions of that time on EHRs outside of scheduled hours and on inbox activities, with median WOW-EHR and IB-EHR ratios of 0.05 (IQR 0.03-0.07) and 0.06 (IQR 0.05-0.08), respectively.

#### “Work Outside of Work” Cluster

“Work outside of work” cluster physicians, constituting 27.2% (47/173) of the total, spent the most time on EHRs (EHR-Time_8_: median 6.83, 5.95-8.36) and the largest proportion of that time outside of work hours (WOW-EHR: median 0.21, IQR 0.17-0.26). This cluster of physicians had average median Note-EHR and Order-EHR ratios of 0.31 (0.27-0.36) and 0.16 (IQR 0.14-0.18), respectively, and an above-average median IB-EHR ratio of 0.15 (IQR 0.11-0.17).

#### “Notes Outside of Work” Cluster

“Notes outside of work” cluster physicians, constituting 19.7% (34/173) of the total, had the second-highest median WOW-EHR ratio of 0.13 (IQR 0.10-0.19) but had near-average total normalized EHR time (EHR-Time_8_: median 5.90, IQR 5.37-6.36). This cluster of physicians had an above-average median Note-EHR ratio of 0.37 (IQR 0.33-0.40) and below-average median IB-EHR and Order-EHR ratios of 0.10 (IQR 0.08-0.12) and 0.14 (IQR 0.12-0.17), respectively.

### Phenotype Clusters by Specialty and Gender

Physician distribution across phenotype clusters by specialty and gender are reported in Table 3. There was a significant association between the clusters and specialty (*X*^2^_6_=26.67, *P*<.001). Pediatricians primarily fell into the “Higher note time” and “Notes outside of work” clusters (16/20, 80%) and accounted for 47% (7/15) of the total physicians in the “Higher note time” cluster. Family and internal medicine physicians were primarily distributed across the “Lower EHR time” and “Work outside of work” clusters (family medicine: 29/36, 81%; internal medicine: 87/113, 77%). In addition, there was a significant association between gender and clusters (*X*^2^_3_=18.28, *P*<.001). Female physicians were more prominent in the “Work outside of work” and “Notes outside of work” clusters, accounting for 64% (30/47) and 62% (21/34) of the clusters, respectively. Male physicians accounted for 71% (52/73) of the “Lower EHR time” cluster.

**Table 3:** Physician specialty and gender distribution by phenotype cluster.

Distribution		Number of physicians (N=173), n (%)	Phenotype clusters					*P* value	
			Lower EHR^a^ time (n=73), n (%)	Higher note time (n=15), n (%)	Work outside of work (n=47), n (%)	Notes outside of work (n=34), n (%)			
**Specialty**									<.001
	Family medicine	36 (21)	19 (26)	2 (13)	10 (21)	5 (15)			
	Internal medicine	113 (65)	52 (71)	6 (40)	35 (74)	20 (59)			
	Pediatrics	20 (12)	2 (3)	7 (47)	2 (4)	9 (26)			
**Gender**									<.001
	Female	80 (46)	21 (29)	8 (53)	30 (64)	21 (62)			
	Male	89 (51)	52 (71)	7 (47)	17 (36)	13 (38)			
Total		169 (98)	73 (42)	15 (9)	47 (27)	34 (20)			

^a^EHR: electronic health record.

## Discussion

### Principal Findings

In this unsupervised clustering machine learning analysis of a cohort of primary care physicians, we identified 4 distinct EHR use phenotypes characterized by the total time spent on EHR activities and the ratios of those times in comparison to one another. These phenotypes were differentiated and described by patterns of use consistent with overall efficiency, higher documentation time, and working outside of work hours; each of these patterns of use were generally associated with the “Lower EHR time,” “Higher note time,” and “Work/Notes outside of work” clusters, respectively. While exploratory, these results provide insights into EHR use phenotypes across gender and specialties that can complement and provide additional context for current EHR use research.

### Work Outside of Scheduled Hours

We identified 2 phenotype clusters that had above-average ratios for work outside of scheduled hours. Although “Work outside of work” and “Notes outside of work” clusters both had high WOW-EHR ratios, only the “Work outside of work” cluster had significantly higher than average EHR-Time_8_. A possible explanation for this is that physicians in the “Work outside of work” cluster work from home partly out of necessity because they require more time on EHRs, whereas physicians in the “Notes outside of work” cluster may elect to finish work at home, suggesting a preference for ad hoc work hours.

### Note Time

Time spent on clinical documentation accounted for the largest proportion of total EHR time in each cluster. There was, however, considerable variation in the ratio of note time to EHR time across clusters: from 0.24 of EHR time in the “Lower EHR time” cluster to 0.46 in the “Higher note time” cluster despite similar total EHR time in both clusters. Potential explanations for this variation include differences in clinic- or physician-specific workflows (eg, scribe support or team-based documentation; differences in depth and complexity of encounters and expectations for documentation; and use of form, copied, or auto-populated notes) and differences in documentation style, particularly among the “Higher note time” cluster that may include physicians who deliberately spend more time on documentation.

### Limitations

This exploratory work only used time-based metrics and did not account for patient acuity or complexity. Although the data were gathered over a 2-year period, systemic differences in patient volume and care could have affected the results. In addition, this work was limited to a single ambulatory practice network in one region of the United States and was limited to primary care physicians. Some types of EHR activities (eg, chart review) were not included in the metrics, and it is possible that other activities or practice domains could also affect clustering. Furthermore, it should be noted that this study only identified EHR use phenotypes and did not explore reasons behind differences in EHR use or assign value to the phenotypes.

### Conclusions

Our findings may highlight opportunities for interventions to improve EHR design and use to better support EHR users’ needs. Potential differences in users’ needs were identified for each phenotype cluster. The “Higher note time” and “Notes outside of work” clusters might benefit from scribe support more than the other two clusters. The “Work outside of work” cluster might benefit from inbox support and restructuring their practice for a more team-based approach. Physicians in the “Lower EHR time” cluster could be consulted as local champions to help their peers improve their EHR efficiency. By identifying and classifying individual EHR use and user needs, we can better understand and target interventions at the individual or department level. Future work should validate these phenotypes in larger cohorts and in diverse settings, explore differences in physicians’ training and demographics across phenotypes, and investigate the relationships among EHR use phenotypes, patient outcomes, and clinician satisfaction and burnout.

## Abbreviations

EHR: electronic health record
EHR-Time_8_: time a physician spends on EHRs normalized to 8 hours of scheduled patient time
IB-EHR: percent of EHR time that a physician spends on inbox activities
IB-Time_8_: inbox time hours normalized to 8 hours of scheduled patient time
Note-EHR: percent of EHR time that a physician spends on documentation
Note-Time_8_: note time hours normalized to 8 hours of scheduled patient time
Order-EHR: percent of EHR time that a physician spends on orders
Order-Time_8_: order time hours normalized to 8 hours of scheduled patient time
WOW_8_: work outside of work hours normalized to 8 hours of scheduled patient time
WOW-EHR: percent of EHR time that occurs during work outside of scheduled hours
